# Loss of *mpv17* affected early embryonic development via mitochondria dysfunction in zebrafish

**DOI:** 10.1038/s41420-021-00630-w

**Published:** 2021-09-18

**Authors:** Wan-Ping Bian, Shi-Ya Pu, Shao-Lin Xie, Chao Wang, Shun Deng, Phyllis R. Strauss, De-Sheng Pei

**Affiliations:** 1 Chongqing Institute of Green and Intelligent Technology, Chinese Academy of Sciences, 400714 Chongqing, China; 2 School of Public Health and Management, Chongqing Medical University, 400016 Chongqing, China; 3 Department of Biology, College of Science, Northeastern University, Boston, MA 02115 USA

**Keywords:** Skin cancer, Gene expression

## Abstract

*MVP17* encodes a mitochondrial inner-membrane protein, and mutation of human *MVP17* can cause mitochondria DNA depletion syndrome (MDDS). However, the underlying function of *mpv17* is still elusive. Here, we developed a new mutant with *mpv17* knockout by using the CRISPR/Cas9 system. The *mpv17*^−/−^ zebrafish showed developmental defects in muscles, liver, and energy supply. The *mpv17*^−/−^ larvae hardly survived beyond a month, and they showed abnormal growth during the development stage. Abnormal swimming ability was also found in the *mpv17*^−/−^ zebrafish. The transmission electron microscope (TEM) observation indicated that the *mpv17*^−/−^ zebrafish underwent severe mitochondria dysfunction and the disorder of mitochondrial cristae. As an energy producer, the defects of mitochondria significantly reduced ATP content in *mpv17*^−/−^ zebrafish, compared to wild-type zebrafish. We hypothesized that the disorder of mitochondria cristae was contributed to the dysfunction of muscle and liver in the *mpv17*^−/−^ zebrafish. Moreover, the content of major energy depot triglycerides (TAG) was decreased dramatically. Interestingly, after rescued with normal exogenous mitochondria by microinjection, the genes involved in the TAG metabolism pathway were recovered to a normal level. Taken together, this is the first report of developmental defects in muscles, liver, and energy supply via mitochondria dysfunction, and reveals the functional mechanism of *mpv17* in zebrafish.

## Introduction

*MVP17* encodes proteins with four-transmembrane spanning in the mitochondrial inner membrane, but its function was not well understood. Different health problems were reported in multiple species. In mice, *Mpv17* was identified early in 1990, and the defect of *Mpv17* was generated by the insertional inactivation of recombinant retrovirus. The homozygotes of *Mpv17*^−/−^ mice were found with health problems like nephrotic syndrome and chronic renal failure [1]. Subsequently, more works were reported on the mouse *Mpv17* defect [2–7], implying that the function of the *Mpv17* gene was involved in the ROS production and glomerulosclerosis. Due to the morphological degeneration of cochlea [5, 6], the abnormality of the mouse inner ear was also reported after the loss of *Mpv17*, which was similar to human Alport syndrome [3, 4]. Clozel et al. [7] revealed that *Mpv17*^*−/−*^ mice developed significant systemic hypertension and tachycardia, and followed by polyuria and elevated natriuresis, indicating the key role of *Mpv17* in the mouse.

In humans, *MVP17* mutation caused mitochondria DNA depletion syndrome (MDDS) and led to death in infancy [8–13]. The clinical symptoms of *MVP17* mutation were hepatopathy, peripheral neuropathy, and failure to thrive [14]. Wong et al. [15] reported that mutations of the *MVP17* gene were responsible for rapidly progressive liver failure in infancy. Most of the human MDDS clinical cases were point mutant or deletion of the *MVP17* [16, 17].

In 2013, the *mpv17* defect in zebrafish was reported, and the phenotype of *transparent* (*tra*) zebrafish was characterized, whose *mpv17* was a deletion resulting in a premature stop codon [18]. The *tra* mutant zebrafish showed a transparent phenotype with iridophore and melanophore defects. In 2017, D’Agati and colleagues provided genetic evidence to roy orbison (*roy*) and *casper* zebrafish [19, 20]. They indicated that a 19 bp deletion in the *mpv17* mRNA caused the line *roy* [21]. They showed no other phenotype in the *roy* zebrafish, implying that there were no obvious defects in zebrafish liver, kidney, and muscle except the major defects of the skin color, compared to humans or mice mutant.

In this study, we developed a newly established zebrafish line with a truncate *mpv17* to show a strong phenotype lacking iridophores and melanophores like *tra* and *roy*. Besides, our *mpv17*^−^^/^^−^ zebrafish hardly survived more than 4 weeks; they had abnormal muscle and liver. The defects were consistent with human and mouse mutants but not the same as the zebrafish mutant line *tra* and *roy*. The mitochondria in the *mpv17*^−/−^ possessed cristae defects, which is the key role for cellular respiration. Thus, the contents of TAG and ATP were decreased in the *mpv17*^−/−^ zebrafish. Our work provided more details and evidence of the relationship between *mpv17* and the development of muscles and the liver. The malfunction mitochondria played a key role in connecting the gene and these abnormal phenotypes. This newly established zebrafish mutant line could help us to elucidate the molecular mechanism of the function of the *mpv17* gene on the MDDS.

## Results

### The generation of the *mpv17*^−/−^ zebrafish

**Fig. 1:**
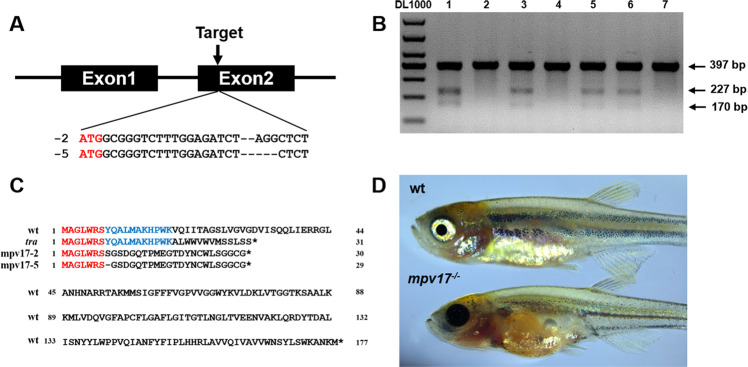
The zebrafish *mpv17* gene was knocked out using the CRISPR/Cas9 system. **A** The targeted loci were near the translation start code ATG of the gene *mpv17*. **B** The agarose gel electrophoresis results of the T7E1 assay for the screening of *mpv17* knocked out zebrafish. The marker is DL1000 and the lane number shows different samples. The 397 bp band indicates the wild-type gene, and the lanes with 227 and 170 bp bands show the mutated genes. **C** The amino acid alignment of the *mpv17* protein sequence in wild-type, *tra* mutant, and *mpv17*^**−/−**^ zebrafish in this study. D One-month-old wild-type and *mpv17*^*−/−*^ zebrafish larvae.

We knocked out the zebrafish *mpv17* using the CRISPR/Cas9 system to generate the *mpv17*^*−/−*^ zebrafish. The target sites were designed at the second exon of the chromosome near the translation start code with an online tool ZIFIT Targeter (http://zifit.partners.org/ZiFiT/ChoiceMenu.aspx) (Fig. 1A). The CRISPR/Cas9 system worked well for knocking out gene *mpv17*, which was confirmed by the T7E1 assay (Fig. 1B). The larvae were raised to adults for screening the homozygous mutant. Two mutants *mpv17*-2 and *mpv17*-5 were identified, which indicated that there were 2 and 5 bp deletions of the gene. For the details of mutants information, the sequences of these mutants were compared with those of wild-type and mutant transparent *(tra)* [18] (Fig. 1C). In the *tra* mutant, a deletion of 54–72 bp in *mpv17* produced a frameshift following a stop code after 31 codons. The N-terminal 18 amino acid residues were identical with the wild-type. In our work, the deletion of 2 and 5 bp in the position 22 bp results in a frameshift and premature stop code. A 30 and 29 amino acid residues were left, and only 7 residues in the N-terminal were identical to the wild-type. Since the *mpv17*-5 showed only one residue less than *mpv17*-2, we performed all the following experiments using the mutant *mpv17*-5, which refers to *mpv17*^−/−^. The 1-month-old *mpv17*^−/−^ zebrafish showed lack of iridophores and melanophores in the belly, dorsum, and eyes, which was similar to the *tra* mutant (Fig. 1D) [18]. The development stages of the wild-type and *mpv17*^*−/−*^ larvae were recorded from zygotes to larvae at 9 days post-fertilization (dpf) (Supplementary Fig. S1). At 5 dpf, the eyes’ reflection of *mpv17*^*−/−*^ larvae were significantly reduced due to the lack of iridophores (Supplementary Fig. S1E, F, G, H, Eʹ, Fʹ, Gʹ, Hʹ).

### Poor growth and low survival beyond 4 weeks in the *mpv17*^−/−^ zebrafish

**Fig. 2:**
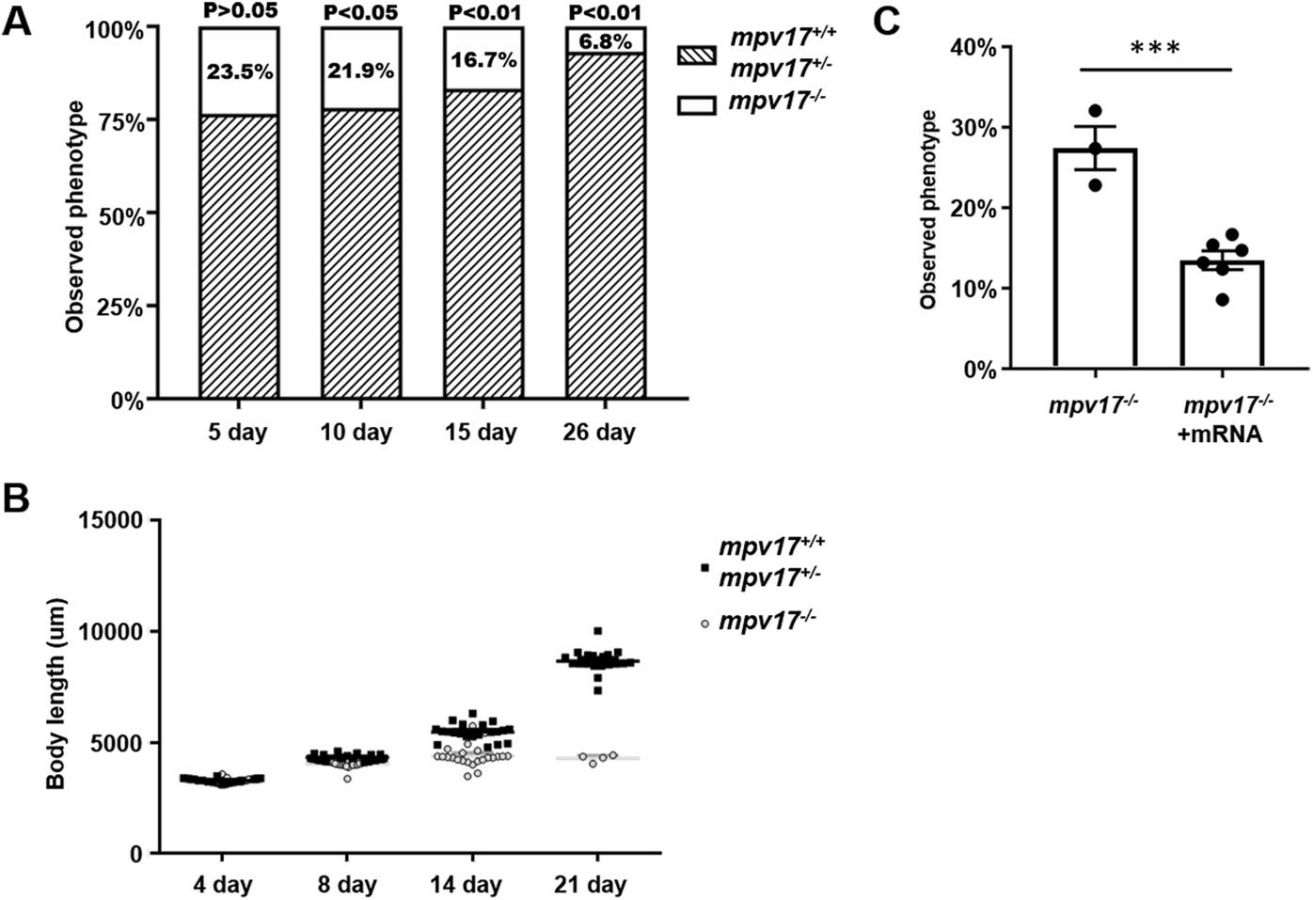
The *mpv17*^*−/−*^ zebrafish has a poor growth condition and low survival ratio, but can be recovered by mpv17 mRNA. **A** The *mpv17*^**−/−**^ zebrafish exhibited a decreased survival ratio in the first month. *n* = 1160 for 5 dpf, 993 for 10 dpf, 227 for 15 dpf and 176 for 26 dpf. **B** The growth of the *mpv17*^**−/−**^ zebrafish was decreased and even stopped after 14 days. The difference in the average length between *mpv17*^**−/−**^ and the pooled (*mpv17*^**+/+**^ and *mpv17*^**+/−**^) larvae was increased. **C** The *mpv17* mRNA can rescue the *mpv17*^−/−^ zebrafish, and the proportion of *mpv17*^−/−^ zebrafish in the rescue group was less than the control. Error bars were indicated with ±SEM, ***p < 0.005.

The heterozygotes *mpv17*^+/−^ zebrafish were crossed, and the offspring eggs were used to check the survival rate assay by calculating the predictable proportion of *mpv17*^−/−^. We pooled *mpv17*^+/+^ and *mpv17*^+/−^ for the survival calculation due to the *mpv17*^+/−^ zebrafish was unable to distinguish from the *mpv17*^+/+^ zebrafish in the phenotype. The *mpv17*^−/−^ zebrafish can be selected according to the reflection color of eyes at 5 days, but there was no difference between *mpv17*^+/+^ and *mpv17*^+/−^. The proportion was no significant in the larvae at 5 dpf (23.5%, 76.5%). At 10 days, the proportion was significantly different from the predicated, when 21.9% of *mpv17*^−/−^, 78.1% of the pooled samples were recovered. At 26 dpf, only 6.8% *mpv17*^−/−^ zebrafish were still alive (Fig. 2A). The body length was also measured in the *mpv17*^−/−^ and pooled *mpv17*^+/+^ and *mpv17*^+/−^ groups. At the early stage of the development, the length of the *mpv17*^−/−^ larvae was no different from the pooled group. After that, the *mpv17*^−/−^ larvae were shorter than *mpv17*^+/+^ and *mpv17*^+/−^ at 14 and 21 dpf. The *mpv17*^−/−^ larvae almost stopped growing, compared to the 14 and 21 dpf samples (Fig. 2B). It indicated that the *mpv17*^−/−^ zebrafish had poor growth and even stopped after 2 weeks. We did the rescue of the *mpv17*^−/−^ larvae using the *mpv17* mRNA injected into the eggs. The proportion of *mpv17*^−/−^ larvae was significantly less in the rescue group, which indicated that the *mpv17* mRNA could recover the phenotype of *mpv17*^−/−^ mutant larvae (Fig. 2C).

### Aberrant skeletal muscle in the *mpv17*^−/−^ zebrafish

**Fig. 3:**
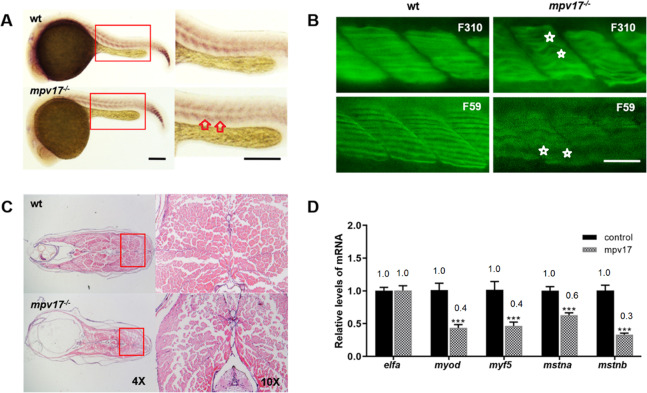
The skeletal muscle showed defects during the development of the *mpv17*^−/−^ zebrafish. **A** In situ hybridization analysis of myogenic marker gene *myod* at 24 hpf. Scale bar = 250 μm. **B** Confocal image of fast and slow muscle at 72 hpf. Antibodies F310 and F59 were used for staining the fast and slow muscle fiber, respectively. Scale bar = 50 μm. **C** Paraffin section and H&E staining analysis of the skeletal muscle structure in the 1-month-old fish. **D** The relative mRNA expression levels of the genes involved in the development of muscles.

During the raising and screening of the homozygotes, we found an obvious phenomenon of the *mpv17*^−/−^ zebrafish with abnormal movements (Supplementary Movie S1). According to the movie, we recorded the swimming behavior of the larvae with different color lines (Supplementary Fig. S2A). We counted the swing times of the caudal fin and the swimming distance (Supplementary Fig. S2B, C). Interestingly, the *mpv17*^−/−^ larvae had long swimming distances for 15 s, and the swing times of caudal fin were significantly higher than those of the wild-type larvae. We considered that the muscle with defects was contributed to this phenotype. Thus, we performed in situ hybridization with the RNA probe for the muscle development marker gene *myod*. This gene is required for somatic and early cranial myogenesis, and some early pectoral fin development [22, 23]. The result showed that the signal of the *myod* was reduced in the *mpv17*^−/−^ zebrafish (Fig. 3A). We confirmed this using qRT-PCR with more genes involved in the muscle development, and the expression levels of the *myf5* [24], *mstna*, and *mstnb* were significantly decreased in the *mpv17*^−/−^ zebrafish (Fig. 3D). For more details of the muscle development, we performed the whole-mount immunofluorescence with antibody F59 and F310, which predominantly recognizes slow muscle myosin fibers and fast muscle myosin fibers. The alterations in the arrangement and integrity of myofibers were observed apparently. The structure of myofiber was irregular, and the tissue displayed a disorganization pattern in the *mpv17*^−/−^ zebrafish, compared to the wild-type (Fig. 3B). It’s indicated that the early development of skeletal muscle in the *mpv17*^−/−^ zebrafish was influenced by the mutant *mpv17* gene. The histological analysis was performed with hematoxylin-eosin (HE) staining of the section for the trunk skeletal muscle tissue (Fig. 3C). The mass of skeletal tissue was less than that of the control. The structure was loose and there were more interspaces between the myofibers in the *mpv17*^−/−^ zebrafish.

### Altered content of major energy depot and the change of related genes in the *mpv17*^−/−^ larvae

**Fig. 4:**
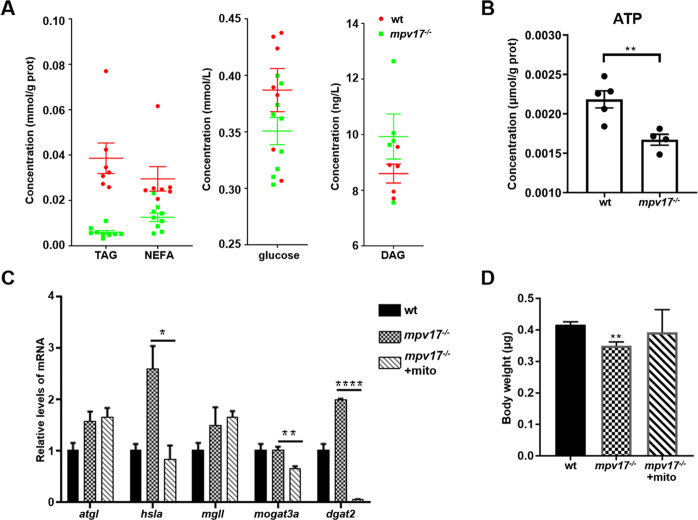
The *mpv17*^−/−^ zebrafish with low contents of TAG and normal mitochondria could rescue the expression of the genes in the TAG metabolism. **A** The contents of the DAG, TAG, NEFA, and glucose were measured at 7 dpf stage. **B** The content of ATP was evaluated at 7 dpf. **C** The relative mRNA expression levels of genes involved in the TAG metabolism in *mpv17*^−/−^ larvae with or without normal mitochondria injection. **D** The bodyweight of the *mpv17*^−/−^ larvae with or without mitochondria injection. Error bars were indicated with ±SEM, *p *<* 0.05, **p *<* 0.01, ****p *<* 0.001.

Due to the motility abnormality of the *mpv17*^−/−^ zebrafish, we wonder whether the energy supply was reduced. Thus, we evaluated the contents of triglyceride (TAG), which is the major energy depot of all eukaryotic cells, and the metabolites diglyceride (DAG) and non-esterified fatty (NEFA) in the *mpv17*^−/−^ larvae. The result showed that the TAG content was significantly reduced in the *mpv17*^−/−^ larvae (0.007 mmol/g prot), compared to the control (0.039 mmol/g prot). For the content of NEFA, the *mpv17*^−/−^ zebrafish was less than the wild-type control. There was no significant difference in the content of DAG between the *mpv17*^−/−^ and control. Moreover, the glucose level was lower in the *mpv17*^−/−^ zebrafish than that of the wild-type (Fig. 4A). These results suggested that the energy substrate of the *mpv17*^−/−^ zebrafish was less than the wild-type. We tried to figure out whether the ATP contents of the *mpv17*^−/−^ zebrafish were disturbed. As expected, the content of ATP in the *mpv17*^−/−^ zebrafish was significantly reduced to half of the wild-type (Fig. 4B). Thus, we checked the expression levels of genes involved in TAG synthesis and degradation. The expression levels of all genes were changed in the *mpv17*^−/−^ zebrafish except *mogat3a*, which can catalyze the first step in triacylglycerol synthesis. The genes *atgl*, *hsla*, and *mgll* that involved in the triglyceride breakdown were significantly increased in the *mpv17*^−/−^ larvae. The increase of the gene expression enhanced the degradation of the triglycerides (Fig. 4C).

Considering the *mpv17* is a gene encoding a protein located in the mitochondrial inner membrane, we tried to figure out whether exogenous mitochondria can recover the gene expression of TAG metabolism or not. We performed the mitochondrial injection to the one-cell stage eggs of the *mpv17*^−/−^. After 7 days, the zebrafish were collected and detected the expression levels of genes involved in triglyceride metabolism. After injection, the expression levels of *hsla* and *mogat3a* were back to the wild-type level, indicating that the exogenous mitochondria may be a good solution to recover the *mpv17* mutant (Fig. 4C). The bodyweight of the *mpv17*^−/−^ larvae at 7 dpf was significantly reduced to approximately 16%, compared to control (Fig. 4D). Interestingly, injection with exogenous mitochondria, the bodyweight of the *mpv17*^−/−^ larvae was also recovered closed to the wild-type level (Fig. 4D).

### Delayed liver development and abnormal tissue

**Fig. 5:**
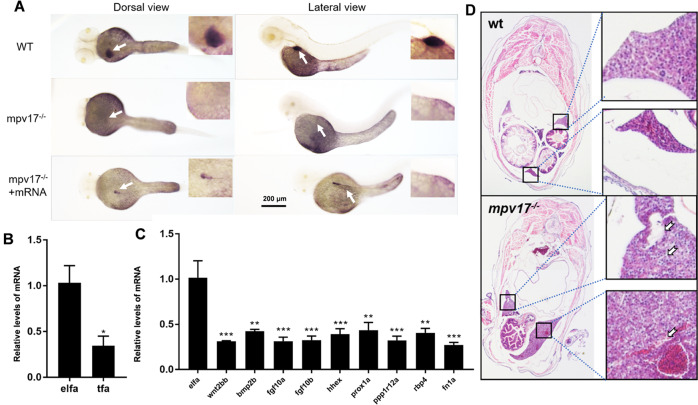
The development of the liver was abnormal in the *mpv17*^*−/−*^ zebrafish. **A** In situ hybridization with RNA probe *tfa* at 3 dpf. Wild-type was in the upper, *mpv17*^−/−^ in the middle, and the lower was the *mpv17*^−/−^ larvae rescued with *mpv17* mRNA. **B** In situ hybridization of larvae at 5 dpf using *tfa* probe. **C** The expression level of genes *tfa*. Error bars were indicated with ±SEM, *p *<* 0.05. **D** The expression levels of genes involved in liver development. Error bars were indicated with ±SEM, **p *<* 0.01, ***p *<* 0.005. **E** H&E staining paraffin-embedded sections of the liver in 1-month-old zebrafish. The black frames were enlarged as shown on the right.

We performed the whole-mount in situ hybridization (WMISH) to evaluate the liver development of the *mpv17*^−/−^ zebrafish with an RNA probe *tfa* (hepatogenic marker). The *tfa* gene signal was strong and presented on the left side of the body in the wild-type larvae from the dorsal view. However, the staining of the probe was absent in the *mpv17*^−/−^ larvae at 3 dpf (Fig. 5A, upper and middle panel). We injected the *mpv17*^−/−^ larvae with *mpv17* mRNA to rescue the phenotype. After 3 days, the *mpv17*^−/−^ larvae had a slight signal using the probe in the same position (Fig. 5A, lower panel). We also performed the experiment with larvae at 5 dpf, and the staining intensity was increased and spread from left to the right side in wild-type larvae from dorsal view. But, the *mpv17*^−/−^ larvae had weak signals on the left side of the liver (Fig. 5B), implying that the development of liver in *mpv17*^−/_^ larvae was delayed, compared to the wild-type. We also performed the qRT-PCR to analyze the expression levels of the genes involved in liver development. The *wnt2bb*, *bmp2b*, and *fgf* play key roles as signaling molecules in the specification of hepatoblasts. The *hhex* and *prox1* can promote liver budding and hepatoblasts migration, respectively [25]. The *ppp1r12a* mediates the liver primordia to promote liver bud growth [26]. The *rbp4* plays a role in the formation of a liver bud, which requires the migration of liver progenitor cells [27]. All genes related to the initial development of the liver reduced significantly in the *mpv17*^−/−^ larvae (Fig. 5C, D). These results confirmed that liver development was delayed in the *mpv17*^*−/−*^ zebrafish. Further, the paraffin section and HE stain were used to check the structure of liver tissue with 1-month-old *mpv17*^−/−^ zebrafish. As shown in Fig. 5E, the liver tissue was discrete and abnormal, compared to the control.

### Hollow mitochondrial cristae and reduced number of mitochondria in the *mpv17*^−/−^ zebrafish

**Fig. 6:**
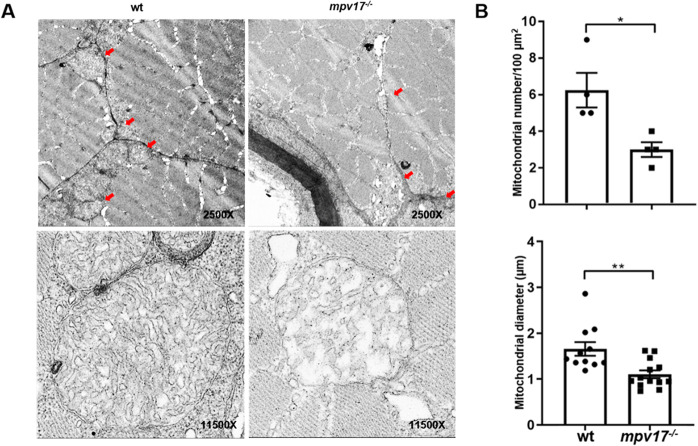
The mitochondria structure, number, and size in the *mpv17*^*−/−*^ larvae muscle. **A** TEM image of the skeletal muscle for the larvae at 7 dpf. The magnification was ×2500 in the upper panel and ×11,500 in the lower panel. Red arrowhead indicated the mitochondria in the skeletal muscles. **B** The number of the mitochondria per 100 μm^2^ (upper) and the diameter of the mitochondria in the skeletal muscle (lower). Error bars were indicated with ±SEM, *p < 0.05 and **p < 0.01.

The muscle tissue of *mpv17*^−/−^ zebrafish at 7 dpf was collected for the mitochondria analysis by TEM. The mitochondria numbers in the *mpv17*^−/−^ larvae muscles were significantly decreased, compared to the wild-type larvae (Fig. 6A, upper panel, Fig. 6B, upper panel). In each 100 μm^2^ of the tissue, the average number of mitochondria was about 6 and 3 in the wild-type and *mpv17*^−/−^ larvae, respectively. The size of the mitochondria in *mpv17*^−/−^ larvae was smaller than that of the wild-type (Fig. 6B, lower panel). The mitochondria were shrunk about 30% of the size in the *mpv17*^−/−^ line. We also saw the hollow mitochondria with broken cristae in the *mpv17*^−/−^ larvae muscle (Fig. 6A). These defects dramatically decreased the surface area of the inner-membrane system, which is the most important place for cellular respiration. Thus, the low content of ATP may be due to the defects of the mitochondrial cristae.

## Discussion

In this study, the *mpv17* knockout zebrafish was generated using the CRISPR/Cas9 system. The *mpv17*^−/−^ zebrafish showed developmental defects in muscles, liver, and energy supply via mitochondria dysfunction, which is similar to human MDDS. The clinical symptoms of human MDDS contain hepatopathy, peripheral neuropathy, failure to thrive, and rapidly progressive liver failure in infancy [14, 15]. In the previous study, a *tra* mutant was first reported in 1983 by Walker and Streisinger [28]. In 2013, this mutant line was well studied in phenotypic and molecular characterization by Krauss [18]. In this *tra* zebrafish, the iridophores and melanophores were significantly reduced due to the mutant of the *mpv17* gene. In 2017, another work had figured out that the *roy* zebrafish has a 19 bp deletion of the *mpv17* mRNA using the positional cloning method. They confirmed it by a rescue experiment with *mpv17* mRNA [21]. However, In these works, the authors found that the mutant of *mpv17* contributed to the zebrafish lacking iridophores and melanophores. Strikingly, they did not find any other defects in the liver, kidney, or muscle except the defects to the skin.

In this study, the homozygote hardly survived 1 month as indicated in the “Result” section, and the developments of liver, muscle, and mitochondria were all abnormal. Our finding was similar to the phenotype of *mpv17* mutant mice that carried an insertion in the *mpv17* gene and destroyed protein function. The *mpv17* mutant mice developed mitochondrial DNA depletion, late-onset glomerulosclerosis, hair graying, and the neurodegeneration of the peripheral nervous system [1, 4, 5, 29]. In the humans, many mutations of this gene had been reported, and the *mpv17* mutation was associated with mitochondrial DNA depletion syndrome and caused death during early childhood [17, 30, 31]. The different phenotypes between our mutant zebrafish and the *tra* or *roy* lines possibly because of the different truncated *mpv17* proteins. In our work, the *mpv17* protein had seven amino acid residues that were identical to the wild-type protein, while the *tra* had 18 amino acids. The 11 amino acids difference perhaps contributed to the diverse phenotype between these mutant lines. Another possible reason was that the deletion way of the gene was different. The *mpv17*^−/−^ zebrafish in this work was established with the deletion of exon 2 using the CRISPR/Cas9 system, and this deletion was introduced by the non-homologous end joining (NHEJ). In the *tra* and *roy* zebrafish, the genotype was caused by a deletion of intron 2, following a cryptic splice donor site in exon 2 [21].

Compared to the wild-type, the movement was abnormal in the *mpv17*^−/−^ zebrafish (Supplementary Movie S1, Supplementary Fig. S2). We considered that the abnormal movement was due to the defects of muscle tissues in the *mpv17*^−/−^ zebrafish (Fig. 3B, C). Further, the number and size of muscle mitochondria were decreased, compared to the wild-type (Fig. 6). The phenomenon may come from mitochondrial remodeling, which is important in the early development of muscle in zebrafish [32]. In this study, we analyzed the mtDNA number of the whole zebrafish and did not find a significant difference between the *mpv17*^−/−^ and wild-type larvae (data not shown). The potential reason was that the mtDNA number was decreased in a specific tissue, and the difference of the whole body was not obvious [33]. However, in the muscle tissue, the mitochondrial number was approximately 50% in the *mpv17*^−/−^ zebrafish, compared to the control (Fig. 6B, upper panel). Besides, the disruption of the mitochondrial cristae was observed in the *mpv17*^−/−^ zebrafish. Similarly, the loss of the cristae [13], dilated cristae [34], and unstructured cristae [35] were found in the liver of MVP17 mutant patients. Further, the *mpv17* plays a vital role in maintaining mitochondrial structure and functionality [36]. The insufficient normal mitochondria with less energy ATP (Fig. 4B) fail to function well. Thus, the muscle fibers are developed irregularly, which may be due to the dysfunction of mitochondria.

Interestingly, the *mpv17*^*−/−*^ larvae with weak muscles showed longer swimming distances and faster caudal fin swing (Supplementary Fig. S2B, C). Due to the defects of the muscles, the angle of caudal fin swing in the mpv17^−/−^ is smaller than that of the wild-type. Thus, they need to move the caudal fin more times to keep balance in the water. The energy consumption for each caudal fin swing is lower. In other words, the mpv17^−/−^ larvae have not enough energy for a longer distance of swing the fin each time. They swim in the water to a long distance by rapidly moving the caudal fin. A previous study showed that a 25-year-old male patient showed weakness and atrophy of both distal muscles and the intrinsic muscles due to the mutation of MPV17 [37]. Another clinical case of MPV17 mutation was reported in a 21-year-old male, who walked only with a slow unsteady gait due to the muscle wasting of hands and distal lower limbs [38]. In the mouse model, the *mpv17*^−/−^ mice described by Viscomi indicated that a subclinical mitochondrial myopathy occurred in a 1-year-old *mpv17*^−/−^ mouse [29], whose muscle tissue had only 25% mtDNA compared to the normal littermates. In the mitochondria, dysfunction of ATP synthase caused the deficiency of the flat lamellar cristae and the formation of separate vesicles with few or no cristae [39–41]. Thus, we hypothesized that the number of mitochondria and the defects in mitochondrial cristae contributed to the lower ATP content, which affected the muscle development and the movement of the *mpv17*^−/−^ zebrafish.

Although the genetic difference exists between zebrafish and human, zebrafish is a good model for the studies of human diseases. The phenotype of *mpv17*^−/−^ zebrafish is similar to human MDDS, because we knocked out *mpv17* gene by deleting 5 bp coding sequences to destroy its function in zebrafish. Our *mpv17*^−/−^ fish has defects in muscles, liver, and energy supply. We did not do the morpholino experiment for knocking down *mpv17*. D’Agati reported that ATG morpholino knockdown of *mpv17* led to a strong *roy* phenocopy with loss of iridophores and visible pericardial edema [21].

In this study, the result of WMISH with probe *tfa* showed less signal intensity in the *mpv17*^−/−^ larvae at 3- 5 dpf (Fig. 5A, B). Especially in the 3-day samples, the staining intensity almost disappeared. The low expression level of *tfa* gene, a hepatic function marker secreted by hepatocytes [42], implied that the secretory *tfa* proteins were suppressed in the hepatocytes. Moreover, the expression levels of the genes involved in liver development were all significantly reduced in the *mpv17*^−/−^ zebrafish (Fig. 5D). These genes played important roles in the specification and migration of hepatoblasts [25, 43, 44], implying that they possibly reduced the development of hepatoblasts to a mature liver.

Taken together, our newly established *mpv17*^−/−^ zebrafish can mimic the phenotypes of human MPV17 depletion diseases, which is firstly reported in the fish model. Due to the deficiency of mitochondria, abnormal defects of muscles, liver, and energy supply appeared in the *mpv17*^−/−^ zebrafish, indicating that our *mpv17*^−/−^ zebrafish can be applied for the therapy study of mitochondrial disease in the future.

## Materials and methods

### Ethical approval

The animal experiment is approved by the Animal Care and Use Committee of Chongqing, China and by the Institutional Animal Care and Use Committee of Chongqing Institute of Green and Intelligent Technology, Chinese Academy of Sciences (Approval ID: ZKCQY0106), which is performed according to the “Guide for the Care and Use of Laboratory Animals” (Eighth Edition, 2011).

### Zebrafish culture

Adult zebrafish of the AB strain were raised and maintained in an automatic water cycle system at 28 °C with a 14-h light and 10-h dark cycling. The male and female were mated once a week, and the eggs were collected and cultured at 28 °C.

### Generation of the *mpv17*^−/−^ zebrafish

The targeted sequence of zebrafish *mpv17* was designed using an online tool ZIFIT Targeter (http://zifit.partners.org/ZiFiT/ChoiceMenu.aspx). PCR was performed to amplify the target sequence for the transcript template. The *mpv17* sgRNA was synthesized in vitro with a MAXIscript T7 Kit (Ambion, USA). A linearized plasmid pXT7-Cas9 as a template for producing the Cas9 capped mRNA transcription in vitro using Ambion mMESSAGE mMACHINE T7 Transcription Kit (Ambion, USA). The RNA products were purified according to the manual of the MicroElute RNA Clean-Up Kit (OMEGA, USA), and the quality and concentration were determined by agarose gel electrophoresis and NanoDrop2000 spectrophotometer (Thermo Fisher Scientific, France). Then, the RNAs were stored at −80 °C before use.

Embryos at the one-cell stage were collected and distributed into a petri dish, and 1 nl mixture with Cas9 capped mRNA (300 ng/μL) and *mpv17* sgRNA (30 ng/μl) was microinjected into embryos using a microinjector (Eppendorf, Germany). The injected eggs were incubated and raised in a petri dish at 28 °C. After 48 h, ten eggs were pooled to extract the total DNA, and the T7E1 assay was performed to detect the efficiency of the *mpv17* targeted. The injected embryos were raised to adults for screening the *mpv17*^−/−^ zebrafish.

### Determination of mitochondrial DNA copy number

Absolute quantitative real-time PCR was performed for quantitating the mitochondrial DNA (mtDNA) copy numbers. A T-Vector pMD20 (Takara, Japan) was inserted with a PCR segment of zebrafish *nd1*. The vector molarity was calculated and diluted to 10^2^, 10^3,^ 10^4^, 10^5^, 10^6^, and 10^7^ copies/μl. The genome DNA of 7 dpf wild-type and *mpv17*^−/_^ zebrafish were extracted and diluted to 3 ng/μl. The quantification used the qRT-PCR for targeting the mitochondrial gene *nd1*. The primers are listed in Table 1. Quantitative real-time PCR was performed using the TB Green™ *Premix Ex Taq*™ II (Tli RNaseH Plus) (Takara, Japan) on an ABI 7300 System (PerkinElmer Applied Biosystems, Foster City, CA, USA). Then, the standard curve was plotted as a logarithmic regression with Ct values against the pMD20 copy numbers. The mtDNA copy number was calculated according to the equation given by the standard curve with *r*^2^ > 0.99.

**Table 1:** Primers used in this study.

Gene/Primer name	Accession no.	Forward primer 5ʹ−3ʹ	Reverse primer 5ʹ−3ʹ	Usage
*mpv17-*gRNA	NM_201165.2	TAGGGTCTTTGGAGATCTTATC	AAACGATAAGATCTCCAAAGAC	The mpv17 target
*mpv17-T7E1*		CCGTTTGTCATAATGTGG	CTGCTTAGGGAGGTTTCT	
*mpv17-*RSC		ttcgaattcATGGCGGGTCTTTGGAGATCTTATCA	atctacgtaTCACATCTTGTTGGCTTTCCAGGAG	
*elfa*	AY422992.1	CTTCTCAGGCTGACTGTGC	CCGCTAGCATTACCCTCC	Muscle related genes
*myf5*	AF253470.1	GCAATACTACAGCCTGCCGAT	CACTGCAAACTGGACACTCCT	
*myoD*	NM_131262.2	TTTATGGGCCCAACGTGTCA	TGTGGAAATTCGCTCCACGA	
*mstna*	NM_001004122.2	AACGACAACAACGACGCAAG	TAGGCGCAGGATGCTCAAAA	
*mstnb*	NM_131019.5	GGAGATATAACGGCGCACCA	TGCTTGAGTCGGAGTTTGCT	
*atgl*	XM_005174256.4	TCATTTCAACCCGGATGCCA	CAATGAAGAGATGCACGGCG	TAG metabolismgenes
*hsla*	NM_001316725.1	TTGATCTCGCACGTTCTCCG	ACAGGGAGAGTTGAGGACCC	
*mgll*	NM_200297.2	GGGATCCCAAACAGGTGGAG	AAAGGGCCACCTGATGTCTG	
*mogat3a*	XM_021470057.1	GTTGGACGTCCCATTCCTGT	GATGTTTGTCGTCCGCGATG	
*dgat2*	NM_001030196.1	CTGGAAACTTTCGCTTGCCC	ATCACGATGACCACAGCGTT	
*wnt2bb*	NM_001044344	GCTGGTCGGGTCTGTAACAA	TTGGTGATGCGTTTTGAGCG	Liver development
*bmp2b*	NM_131360	CGAGATCGACCGACGGAAAT	GCAAGCGTAGCTCAAACTCG	genes
*fgf10a*	NM_182870.2	TTTCGGCATTGACTGCAAGC	GCGCTCAGACCTACGAACAT	
*fgf10b*	NM_001045858.1	TCACATCGGTGGATGTTGGG	CGGCAATTTGTGCCGTAGTT	
*hhex*	NM_130934.1	CCCCGAACTCCTCTTTCACC	GACAGAACCGGGTGTATCGG	
*prox1a*	NM_131405.2	TTTTTACCCGCGCAAAAGCA	ACGTTGGACTTCTCACCGTC	
*rbp4*	NM_130920.2	GGATGGAACCATGACAGCCA	CAGCAGCTCCCCAGTACTTC	
*ppp1r12a*	NM_001003870.2	CCGTCAAGCGTTTCCAACAG	TGTGCTCACCTTTTTGGTAGGT	
*fn1a*	NM_131520.2	TGCAGTGGAGTACAGCATCAA	GGCTTGTCCACCGTTGTAACT	
*tfa*	NM_001015057	GAACCCGTCAGCACCTACAA	GTAACTTGCGGTCCCTCCTG	
*myod*	NM_131262.2	GAGAAAATCAAGCGCAAAGAAGC	TAATACGACTCACTATAGGAGCCCCATCATAGCCATAATACTG	In situ hybridization
*tfa*	NM_001015057	GTCCACCAACCCGAACCAGA	TAATACGACTCACTATAGGGCCACCGCATAAAGAAAGAAAGAAA	

### Analysis of mRNA expression level

Total RNA was extracted by RNAiso Plus (Takara, Japan) from 20 larvae at 5 or 7 dpf. Then the cDNA was synthesized using a PrimeScript RT reagent kit with gDNA Eraser (Takara, Japan). qRT-PCR was performed with a TB Green™ *Premix Ex Taq*™ II (Tli RNaseH Plus) (Takara, Japan) on an ABI 7300 System (PerkinElmer Applied Biosystems, Foster City, CA, USA). The expression levels of the genes were calculated with the 2^−ΔΔCt^ method. All the primers used in this work are listed in Table 1.

### Histological examination of the *mpv17*^−/−^ zebrafish

H&E staining of the muscle and liver tissue was performed. The 1-month-old *mpv17*^−/−^ and wild-type zebrafish were selected and fixed in 4% paraformaldehyde (PFA) overnight. The samples were embedded in paraffin wax, dehydrated in ethanol, and sectioned into 5 mm slices. Then the paraffin sections of different samples were stained with H&E and photographed under a microscope (Nikon, Eclipse Ti, Japan).

### Whole-mount in situ hybridization

For the WMISH, the embryos at different growth stages were fixed in 4% paraformaldehyde. We did the WMISH according to the protocol described by Thisse [45]. The antisense probe was designed to target gene transcript sequences. A T7 RNA polymerase promoter (T7: 5ʹ-TAATACGACTCACTATAGGG-3ʹ) was included in the appropriate primer for amplifying the probe template. The RNA probes used in this experiment were about 700 bp. The primers were synthesized by GENEWIZ (China) and the sequences are presented in Table 1. The probe templates were amplified and purified by TAINquick Midi Purification Kit (TIANGEN, China) and then transcribed with MAXIscript T7 Kit (Ambion, USA) in vitro. The products were purified by MicroElute RNA Clean-Up Kit (OMEGA, USA) and quantified with NanoDrop2000 spectrophotometer (Thermo Fisher Scientific, France).

### Whole-mount immunofluorescence

The whole-mount immunofluorescence was performed as follows. The 3 dpf zebrafish embryos were fixed in 4% PFA overnight, then washed three times with PDT (1× phosphate buffered saline (PBS), 0.1% Tween-20, 0.3% Triton-X, 1% dimethyl sulfoxide-DMSO). After blocking 1 h at room temperature with blocking buffer (1× PBS, 0.1% Tween-20, 10% fetal bovine serum (FBS), 2% bovine serum albumin (BSA)), the monoclonal antibody F59 (Developmental Studies Hybridoma Bank-DHSB, 1:20) and F310 (DHSB, 1:20) were used to anti-MHC and MLC, respectively. After incubation for 2 h at room temperature, the samples were washed and blocked again, then incubated with Alexa Fluor® 488 Goat Anti-Mouse (IgG) for 1.5 h at room temperature. Finally, the samples were submerged in 90% glycerol and imaged using an Olympus FluoView™ FV1000 Confocal Microscope (Japan).

### Measurement of the contents of TAG, DAG, NEFA, and ATP

Wild-type and *mpv17*^−/−^ larvae at 7 dpf were selected to measure the contents of triglyceride (TAG), diglyceride (DAG), non-esterified fatty acids (NEFA), and adenosine triphosphate (ATP) as described in the manufacturer’s protocol, respectively. For the tissue samples, the samples were washed with cold PBS, then resuspended and homogenized in extraction buffer. After centrifugation with 12,000 rpm at 4 °C, the supernatant was harvested for the following assay according to the manual instruction. The kits were used as follows: triglyceride assay kit and non-esterified free fatty acids assay kit (Nanjing Jiancheng, Nanjing, China), Zebrafish DAG ELISA kit (Mlbio, Shanghai, China), enhanced ATP assay kit (Beyotime, Shanghai, China).

### Rescue of the *mpv17*^−/−^ larvae with *mpv17* mRNA

The full open reading frame (ORF) of zebrafish *mpv17* (NM_201165.2) was amplified using the PrimeSTAR HS Premix (Takara, Japan). Then, the amplicons were inserted into the pCS2+ vector for the in vitro transcription of *mpv17* capped mRNA. We injected the *mpv17* capped mRNA into the one-cell stage embryos from the *mpv17*^+/−^ zebrafish parents at a concentration of 100 ng/μl. The samples were incubated at 28 °C before collection.

### Analysis of the mitochondria structure of zebrafish muscle with TEM

TEM was used for further observation of the mitochondria. The zebrafish larvae at 7 dpf were selected and fixed in 2.5% glutaraldehyde solution, diced into 1 mm^3^, and immersed in 0.1 M phosphate buffer (pH 7.4) overnight. The treated samples were fixed in 1% OsO4 buffer for 1 h at 4 °C. Samples were dried in a graded series of ethanol and then further dehydrated using an epoxy resin. Finally, ultrathin sections (60−80 nm) of tissues were obtained using a Leica EM UC6 ultra-microtome and examined with a Hitachi (HT-7700) TEM at 80 kV. Three individual samples were randomly collected and used for TEM analyses. The number of the mitochondria was counted and calculated as number per μm^2^. The diameter of the mitochondria was measured by the long axis.

### Mitochondria microinjection to the *mpv17*^−/−^ zebrafish embryos

The mitochondria were isolated from wild-type zebrafish liver using a mitochondrial extraction kit (Solarbio, Beijing, China). According to the manufacturer’s protocol, ten wild-type adult zebrafish livers were collected for the isolation procedure. The livers were washed three times with cold PBS, and then homogenized in the extraction buffer. The homogenate was centrifuged at 1000 × *g* for 5 min, and repeated once. Subsequently, the supernatant was centrifuged at 12,000 × *g* for 10 min, and the pellets were washed twice with wash buffer. The pellets were dissolved in PBS and the concentration was determined by BCA assay. Finally, the microinjection of mitochondria was done according to the previous description [46].

### The survival ratio and the body length of *mpv17*^−/−^ zebrafish

The survival ratio of *mpv17*^−/−^, pooled *mpv17*^+/−^, and *mpv17*^+/+^ zebrafish was determined by comparing the observed phenotype with the expected ratio of 1:3. The eggs were harvested from individual crossbreeding of heterozygous parents and reared in beakers. Eggs were collected and the larvae number was counted and recorded. Pearson’s chi-square analyses were used to test whether expected genotype proportions were recovered at each time point. The body length of the zebrafish was measured in each group (*mpv17*^−/−^, pooled *mpv17*^+/−^, and *mpv17*^+/+^). The larvae at 4, 8, 14, and 21 dpf were anesthetized with MS222 (Aladdin, China) and taken pictures for recording the body length.

### Movie record and analysis of the swimming behavior

A movie was made with five of each wild-type and *mpv17*^−/−^ zebrafish larvae. We recorded the swimming trail with different color lines. The swing time of the caudal fin was counted for 15 s, and the swimming distance of each larva was calculated at the same time.

### Statistics analysis

For statistical analysis, one-way analysis of variance (ANOVA) was applied to calculate the differences between the control and experimental groups by GraphPad Prism version 5.01 (GraphPad Software, USA). A value of *p* < 0.05 was considered statistically significant, and all values were expressed as the means ± standard error of the mean (SEM). Each experiment was performed three times independently.

## Supplementary information

SI text

Figure S1

Figure S2

SI Movie

## Data Availability

The authors confirm that the data supporting the findings of this study are available within the article and its supplementary materials.
