# Supplementary material for: Loss of mpv17 affected early embryonic development via mitochondria dysfunction in zebrafish
Source: Cell Death Discov. 2021 Sep 18;7:250. doi: 10.1038/s41420-021-00630-w (PMC8449779; doi:10.1038/s41420-021-00630-w)
Supplement: Supplementary file 1 — SI text [file 41420_2021_630_MOESM1_ESM.docx]

**Supplementary Information**

**Loss of *mpv17* affected early embryonic development *via* mitochondria dysfunction in zebrafish**

Wan-Ping Bian^1^, Shi-Ya Pu^1,2^, Shao-Lin Xie^1^, Chao Wang^1^, Shun Deng^1^, Phyllis R. Strauss^3^, De-Sheng Pei^2*^

^1^Chongqing Institute of Green and Intelligent Technology, Chinese Academy of Sciences, Chongqing 400714, China.

^2^ School of Public Health and Management, Chongqing Medical University, Chongqing 400016, China.

^3^Department of Biology, College of Science, Northeastern University, Boston, MA 02115, USA

^*^Corresponding author.

E-mail: [deshengpei@gmail.com](mailto:deshengpei@gmail.com) and [peids@cigit.ac.cn](mailto:peids@cigit.ac.cn) (D.S.P)

**Figure S1. The development of the zebrafish larvae.** A-H represented the wildtype; A’-H’ represented the mpv17-/-. The figure A, A’ at 1 dpf, B, B’ at 2 dpf, C, C’ at 3 dpf, D, D’ at 4 dpf, E, E’ at 5 dpf, F, F’ at 6 dpf, G, G’ at 7 dpf, and H, H’ at 9 dpf. The scale bar=200 um.

**Figure S2. The movement of the wildtype and the mpv17-/- zebrafish.** A 15 s film was extracted from the movie S1. The motion track was marked with different colors (A), the caudal fin swing times (B), and distance (C) were calculated, respectively.

**Supplementary Movie S1**

**The spontaneous swimming behavior of the *mpv17^-/-^* and the wild-type zebrafish**. The control zebrafish swam smoothly up and down and from left to the right (left). The movement of *mpv17^-/-^* zebrafish was abnormal, and they sank after each swim (right).
