# Supplementary figures and images for: Loss of mpv17 affected early embryonic development via mitochondria dysfunction in zebrafish
Source: Cell Death Discov. 2021 Sep 18;7:250. doi: 10.1038/s41420-021-00630-w (PMC8449779; doi:10.1038/s41420-021-00630-w)

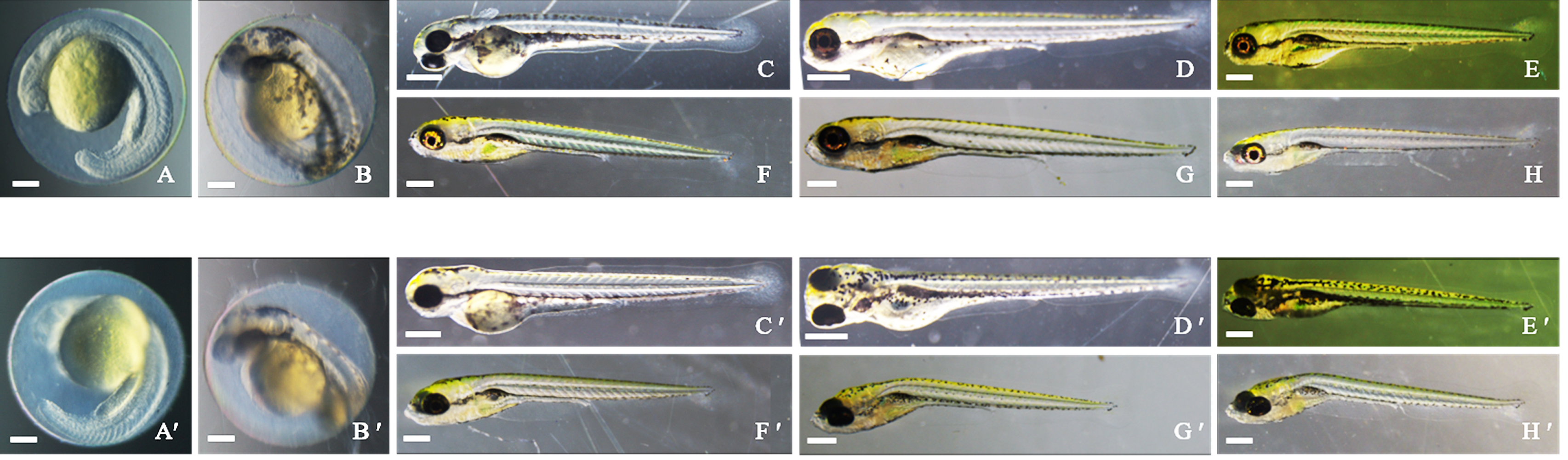

Supplement: Supplementary file 2 — Figure S1 [file 41420_2021_630_MOESM2_ESM.tif]

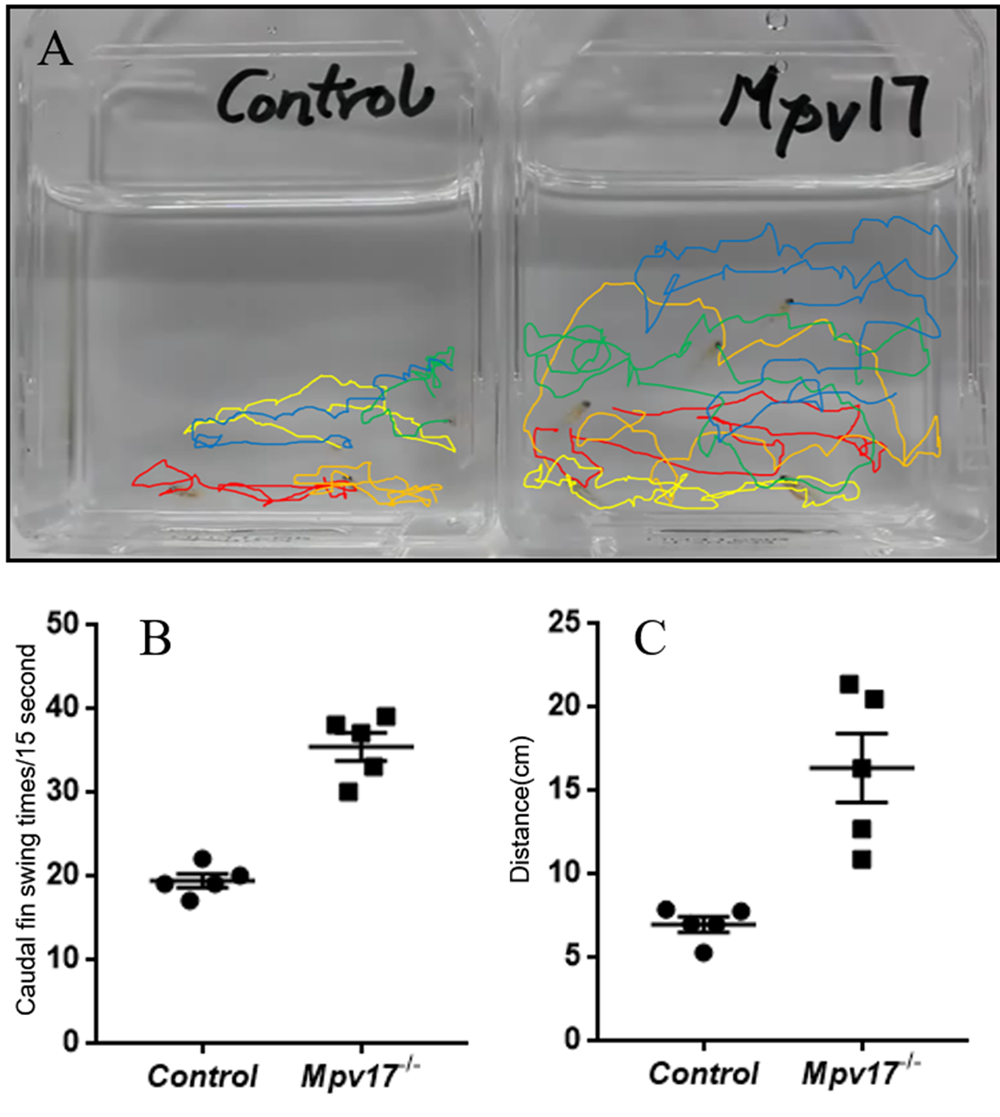

Supplement: Supplementary file 3 — Figure S2 [file 41420_2021_630_MOESM3_ESM.tif]
